# Associations between LDL/HDL-cholesterol ratio and thoracic and abdominal aortic atherosclerosis assessed by MRI

**DOI:** 10.1186/1532-429X-14-S1-P72

**Published:** 2012-02-01

**Authors:** Yukihiko Momiyama, Reiko Ohmori, Ryuichi Kato, Hiroaki Taniguchi, Masayoshi Nagata, Fumitaka Ohsuzu

**Affiliations:** 1 Department of Cardiology, National Hospital Organization Tokyo Medical Center, Tokyo, Japan; 2 National Defense Medical College, Tokorozawa, Japan; 3 Iruma Heart Hospital, Saitama, Japan

## Background

Recently, the LDL/HDL-cholesterol ratio has become recognized as a stronger risk predictor of cardiovascular diseases than each lipid parameter. However, the association between LDL/HDL-cholesterol ratio and aortic atherosclerosis has not been elucidated yet. Using MRI, we investigated the associations between thoracic and abdominal aortic atherosclerosis and LDL/HDL-cholesterol ratio as well as LDL- and HDL-cholesterol levels.

## Methods

Aortic MRI was performed on Signa 1.5T Cvi using a phased-array body coil in 159 patients at high-risk for CAD. However, since statins affect LDL- and HDL-cholesterol levels, 64 patients who were taking statin were excluded from this study. Transverse PDW and T2W images of thoracic descending and abdominal aortas were obtained using an ECG-gated, double-inversion-recovery FSE sequence: TR=2 RR intervals, TE=10ms (PDW) and 60ms (T2W), 20-cm FOV, 4-mm slice thickness, 8-mm inter-slice gap, 256x256 acquisition matrix, and 32 echo-train. For each patient, 9 slices of thoracic aorta and 9 slices of abdominal aorta were obtained at 12-mm intervals, which each covered about 10-cm portion of thoracic aorta below the arch and 10-cm portion of abdominal aorta above the bifurcation of iliac artery. Plaque extent in each slice was scored 0 to 4 points by the percentage of luminal surface involved by plaque. The severity of aortic atherosclerosis was represented as sum of scores (plaque score).

## Results

Among the 95 study patients without statin (age 63±9 years), thoracic and abdominal aortic plaques were found in 60 (63%) and 84 (88%) patients, respectively. The severities of both thoracic and abdominal aortic atherosclerosis correlated with age and blood pressures. Regarding lipid parameters, the severity of thoracic aortic atherosclerosis correlated with LDL-cholesterol (r=0.30) and HDL-cholesterol (r=-0.26) levels (P<0.01) and much better with LDL/HDL-cholesterol ratio (r=0.36, P<0.001). However, the severity of abdominal aortic atherosclerosis correlated with only HDL-cholesterol level (r=-0.29, P<0.005) but not with LDL-cholesterol or LDL/HDL-cholesterol ratio (P=NS). In multiple linear regression analysis, age and LDL/HDL-cholesterol ratio were the independent factors associated with thoracic aortic atherosclerosis, whereas age and HDL-cholesterol level as well as HbA1c and smoking were the factors associated with abdominal aortic atherosclerosis.

## Conclusions

Among lipid parameters, the LDL/HDL-cholesterol ratio was found to be a stronger factor associated with thoracic aortic atherosclerosis than either LDL-cholesterol or HDL-cholesterol levels, whereas abdominal aortic atherosclerosis was associated with only HDL-cholesterol levels. Our results suggest that thoracic and abdominal aortas may have different susceptibilities to atherosclerotic risk factors. MRI is a very useful tool for non-invasively evaluating atherosclerosis in both thoracic and abdominal aortas.

## Funding

Our study had no funding.

